# Optimized planting density and organic fertilizer substitution improve maize yield by enhancing dry matter translocation and grain-filling characteristics

**DOI:** 10.3389/fpls.2026.1913471

**Published:** 2026-08-10

**Authors:** Liping Liu, Yonghui Dai, Wenlong Wang, Yurong Chang, Yining Ma, Risheng Ding, Shaozhong Kang, Ling Tong

**Affiliations:** 1 State Key Laboratory of Efficient Utilization of Agricultural Water Resources, Beijing, China; 2 National Field Observation and Research Station (Gansu Wuwei) for Efficient Water Utilization in Oasis Agriculture, Wuwei, China; 3 Center for Agricultural Water Research in China, China Agricultural University, Beijing, China

**Keywords:** dry matter, grain-filling characteristics, maize yield, organic fertilizer substitution, planting density

## Abstract

**Introduction:**

Increasing planting density and partial substitution of chemical fertilizer with organic fertilizer are important strategies for improving maize yield. However, how their interaction regulates dry matter accumulation, translocation, and grain-filling characteristics to affect yield formation remains unclear.

**Methods:**

A three-year field experiment (2023–2025) was conducted with three planting densities (70,000, 100,000, and 130,000 plants ha^−1^) and four fertilization treatments (no fertilization, chemical fertilizer only, and 17% and 34% organic fertilizer substitution) to evaluate canopy light interception, dry matter accumulation and translocation, grain -filling characteristics, and yield formation.

**Results and discussion:**

The results showed that 17% organic fertilizer substitution improved radiation use efficiency at silking by 7.76%–20.42%. Although increasing planting density reduced per-plant dry matter accumulation by 20.29%–36.53%, 17% organic fertilizer substitution improved dry matter partitioning and partly alleviated the reductions in grain-filling duration and grain weight. Random forest analysis identified aboveground biomass accumulation as the primary predictor of yield and showed that maximum and mean grain-filling rates contributed more strongly to yield prediction than the active grain-filling period. Partial least squares path modeling further revealed that yield reduction under high density was mainly attributed to suppressed post-silking dry matter accumulation, while fertilization promoted dry matter accumulation and translocation, thereby strengthening their positive contributions to grain filling and yield formation. Across three growing seasons, 100,000 plants ha^−1^ with 17% organic fertilizer substitution (D2F2) increased yield by 7.46%–23.83% compared with conventional management (D1F1). Therefore, D2F2 may serve as a promising management option for improving dry matter translocation, grain filling, and maize yield. These results provide valuable references for optimizing density–fertilization management in semi-arid maize production systems.

## 1 Introduction

Maize (*Zea mays* L.) ranks among the world’s three major staple crops, contributing about 40% of total global cereal production (FAOSTAT, 2025), and is indispensable for safeguarding food security, supporting livestock production, and providing industrial feedstocks (Revilla et al., 2022). Achieving high-yield and efficient maize production depends on balancing population structure and nutrient supply, in which planting density and fertilization management serve as two critical regulatory strategies (Lázaro-Rojas et al., 2023). Nevertheless, in current agricultural practices, inappropriate planting densities and overuse of chemical fertilizers are widespread, leading to reduced resource-use efficiency, heightened environmental pollution risks, and limited exploitation of yield potential (Wuepper et al., 2020; Mueller et al., 2012). Consequently, developing cultivation and fertilization strategies that enable high-yield and efficient maize production is of significant practical importance for enhancing agricultural sustainability.

Planting density is a critical determinant of maize yield improvement and functions as a bridge integrating genotype, environment, and management factors (Tian et al., 2024; Wu et al., 2025). Appropriate dense planting improves leaf area index and the interception of photosynthetically active radiation, thereby markedly increasing population dry matter production and yield (Ma et al., 2025; Liu et al., 2022). However, when planting density surpasses the cultivar threshold, intensified competition for light, water, and nutrients results in a disruption of source–sink balance and markedly inhibits individual plant development (Zhang et al., 2024a): On one hand, declines in stem mechanical strength and accelerated leaf senescence shorten the post-silking canopy photosynthetic duration and reduce photosynthetic efficiency (Xue et al., 2020; Jafari et al., 2024); On the other hand, inadequate assimilate supply from source tissues limits the translocation and allocation of post-silking photosynthates to grains, shortens the effective grain-filling period, and ultimately reduces kernel weight per ear (Chen et al., 2021; Wang et al., 2023). Particularly in arid and semi-arid regions, limited water availability intensifies source–sink conflicts under high-density planting, making it a critical scientific challenge to mitigate density-induced stress through integrated agronomic practices for achieving high maize yields.

Regarding nutrient management, partial substitution of chemical fertilizer with organic fertilizer has been increasingly used in maize production to improve both crop yield and soil quality (Liu et al., 2024a; Mbuthia et al., 2015). This integrated fertilization strategy can improve soil aggregate structure (Tian et al., 2022b), stimulate microbial and enzymatic activities, enhance soil water and nutrient retention, and promote root growth and photosynthetic performance, thereby increasing nitrogen uptake and post-silking dry matter accumulation (Liu et al., 2024b; Xin et al., 2017). However, the magnitude of these benefits depends strongly on the proportion of chemical fertilizer replaced by organic fertilizer. An appropriate substitution proportion can improve pre-silking stem dry matter translocation efficiency and its contribution to grain filling while promoting post-silking dry matter partitioning to grains (Liu et al., 2020). However, the appropriate organic fertilizer substitution ratio remains uncertain. Previous studies have reported that substitution ratios of 15%–30% can maintain maize yield (Zhai et al., 2024), whereas a ratio of 50% produced the greatest yield increase in another study (Wang et al., 2019). These contrasting findings indicate that the yield response to organic fertilizer substitution is context-dependent and that a ratio effective under one production condition may not be suitable under another. Identifying a context-specific substitution ratio is therefore necessary to reduce chemical fertilizer input while maintaining adequate nutrient availability and crop yield.

Planting density may be an important source of this context dependency because it determines crop nutrient demand and the balance between individual-plant development and population-level productivity. However, planting density and organic fertilizer substitution have generally been investigated as separate management factors, and it remains unclear whether the appropriate substitution ratio changes with planting density. In particular, how their interaction regulates canopy radiation interception, pre- and post-silking dry matter accumulation and translocation, grain-filling characteristics, and ultimately yield formation remains poorly understood, particularly in semi-arid maize production systems. This unresolved density–substitution interaction represents a key knowledge gap. We therefore hypothesized that an appropriate organic fertilizer substitution ratio could alleviate density-induced constraints on individual-plant development, promote assimilate translocation from vegetative organs to grains, and improve grain-filling characteristics and yield formation. To test this hypothesis, a three-year fixed-site field experiment was conducted with the following objectives: (1) to quantify the responses of canopy radiation characteristics and dry matter accumulation, partitioning, and translocation to planting density and organic fertilizer substitution; (2) to determine how these responses are associated with grain-filling characteristics and yield formation; and (3) to identify the best-performing density–fertilization combination among the treatments evaluated. By integrating canopy radiation, dry matter dynamics, grain-filling characteristics, and yield responses over three growing seasons, this study provides mechanistic evidence for improving density–fertilization management in semi-arid maize production systems.

## 2 Materials and methods

### 2.1 Experimental site

**Figure 1:**
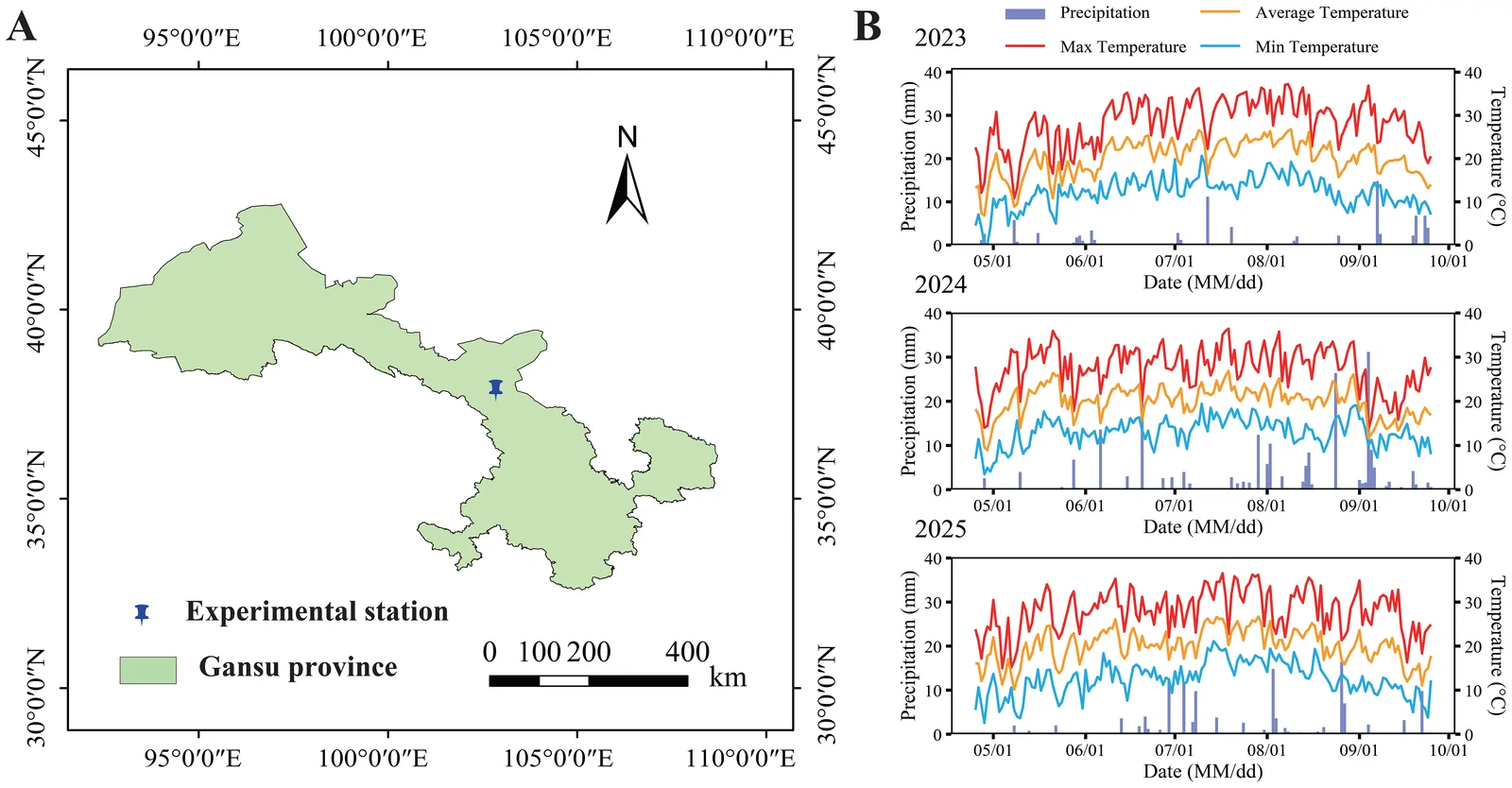
Experimental site and conditions. **(A)** Experimental site. **(B)** Meteorological data during the maize growing seasons from 2023 to 2025.

The field experiment was conducted from 2023 to 2025 at the National Field Observation and Research Station (Gansu Wuwei) for Efficient Water Utilization in Oasis Agriculture, Wuwei, Gansu Province, China (37°52′ N, 102°50′ E; 1581 m a.s.l.) (**Figure 1A**). The study area is characterized by a temperate continental arid climate with abundant solar-thermal resources but scarce precipitation. According to long-term regional climatological records, the mean annual accumulated temperature is approximately 3,550 °C, the annual sunshine duration exceeds 3,000 h, and the annual frost-free period is more than 150 d. The long-term mean annual precipitation and pan evaporation are approximately 182 and 2,200 mm, respectively. The soil is classified as sandy loam according to the USDA textural classification, with a bulk density of 1.51 g cm^−^³, a field capacity of 0.29 cm³ cm^−^³, and a permanent wilting point of 0.12 cm³ cm^−^³. During the experimental period, daily minimum and maximum air temperatures and precipitation were recorded by an on-site automatic weather station (**Figure 1B**). For each experimental year, the meteorological observation window was defined as the maize growing season from sowing to physiological maturity. Cumulative precipitation during this period was 78.6, 200.0, and 115.0 mm in 2023, 2024, and 2025, respectively. The lowest daily minimum air temperatures recorded during the corresponding growing seasons were 4.5, 3.5, and 3.6 °C, whereas the highest daily maximum air temperatures were 37.2, 36.4, and 36.6 °C, respectively.

### 2.2 Experimental design and field management

The experiment was conducted using a randomized complete block design with two factors. The first factor was planting density, comprising three levels: 70,000 plants ha^−1^ (D1), 100,000 plants ha^−1^ (D2), and 130,000 plants ha^−1^ (D3), where D1 represented the conventional local planting density. D1 and D2 were evaluated in all three experimental years, whereas D3 was added in 2025 as an exploratory higher-density treatment. The second factor was fertilization treatment, comprising four levels: no fertilization (F0), chemical fertilizer only (F1), 17% organic fertilizer substitution (F2), and 34% organic fertilizer substitution (F3).

The F2 and F3 treatments corresponded to bio-organic fertilizer application rates of 600 and 1200 kg ha^−1^, respectively. The F2 and F3 treatments received bio-organic fertilizer at rates of 600 and 1200 kg ha^-1^, respectively. Given the N concentration of the bio-organic fertilizer (8.44%), these application rates supplied 50.64 and 101.28 kg N ha^-1^, corresponding to 16.88% and 33.76% of the target N input of 300 kg N ha^-1^, respectively; these values were rounded to 17% and 34% for treatment designation. The two levels were selected to represent low and moderate substitution rates and to provide a twofold gradient in bio-organic fertilizer input. Eight treatment combinations were established in 2023 and 2024, and 12 treatment combinations were established in 2025. Each treatment had three replicates, and each plot measured 15 m × 4.8 m (72 m^2^).

Fertilizer application rates were determined based on local agronomic practices. Except for F0, all fertilized treatments received identical total nutrient inputs of 300 kg N ha^−1^, 87.3 kg phosphorus (P) ha^−1^, and 45.7 kg potassium (K) ha^−1^. Specifically, 87.3 kg P ha^−1^ and 45.7 kg K ha^−1^ are equivalent to approximately 200 kg P_2_O_5_ ha^−1^ and 55 kg K_2_O ha^−1^, respectively. Although the bio-organic fertilizer substitution ratios were calculated on an N-equivalent basis, the P and K supplied by the bio-organic fertilizer were also included in the nutrient balance. The amounts of chemical P and K fertilizers were therefore correspondingly reduced in F2 and F3. Detailed nutrient inputs for each fertilization treatment are presented in **Supplementary Table 3**. The chemical fertilizers used were urea containing 46% N, calcium superphosphate containing 7.0% P, and potassium magnesium sulfate containing 19.9% K. The organic fertilizer was a bio-organic fertilizer developed and manufactured by Ningxia Eppen Biotech Co., Ltd., containing 46.1% organic matter, 8.44% N, 0.42% P, and 0.81% K. All bio-organic fertilizer, P fertilizer, K fertilizer, and 50% of the chemical N fertilizer were applied as a basal dressing and incorporated into the soil before sowing. The remaining 50% of the chemical N fertilizer was applied as urea topdressing at the silking stage.

The spring maize cultivar used was ‘Xianyu 1225’, planted in a configuration of four rows under a single plastic film mulch with two drip lines (**Supplementary Figure 1**). The row spacing was uniformly set at 40 cm, and the intra-row plant spacings for D1, D2, and D3 were 35.7, 25.0, and 19.2 cm, respectively. Subsurface drip irrigation under plastic film mulch was employed, using inline patch-type drip tapes with an emitter spacing of 300 mm, a flow rate of 2.5 L h^−1^, an inner diameter of 16 mm, and a wall thickness of 0.2 mm. All treatments were maintained under a full-irrigation regime. Soil water content was measured at intervals of 10–15 d, and irrigation was applied to restore soil water content to 100% of field capacity. The irrigation amount was adjusted separately for each treatment according to its soil water deficit (Allen et al., 1998). Seven irrigation events were conducted in each growing season. Seasonal irrigation amounts ranged from 492 to 544 mm in 2023, 360 to 413 mm in 2024, and 291 to 390 mm in 2025. Detailed irrigation dates and treatment-specific amounts are provided in **Supplementary Figure 4**. Maize was sown on 2 May 2023, 27 April 2024, and 1 May 2025. Herbicides and insecticides consistent with local practices were applied throughout the experiment to manage weeds and pests.

### 2.3 Sampling and measurements

#### 2.3.1 Canopy radiation interception and radiation use efficiency

The fraction of intercepted photosynthetically active radiation (FIPAR), intercepted photosynthetically active radiation (IPAR), and RUE were determined to evaluate canopy light interception and radiation conversion efficiency. FIPAR was measured at the late-jointing, silking, and grain-filling stages using an AccuPAR LP-80 canopy analyzer (METER Group, USA). The measurements used for the RUE calculations began at late jointing, after canopy closure. For each treatment, a representative 0.80 m × 0.80 m area was selected, and measurements were conducted on clear, cloudless, and windless days between 13:00 and 14:00. Incident photosynthetically active radiation (PAR) above the canopy and transmitted PAR near the soil surface were measured simultaneously. To analyze the vertical distribution of light within the canopy, measurements were performed at two levels: (1) the lower canopy, with the probe positioned close to the soil surface; and (2) the middle canopy, at half of the plant height during the jointing stage and at ear level from silking onward. Multiple readings were taken in a cross-pattern to reduce spatial heterogeneity. FIPAR was calculated using Equation 1 as follows:

(1) $$FIPAR=\frac{PAR_{above}-PAR_{below}}{PAR_{above}}$$

Where *PAR_above_* and *PAR_below_* are incident *PAR* above the canopy and transmitted *PAR* below the canopy, respectively.

Daily incident PAR was obtained from the on-site HOBO automatic weather station between 25 April and 25 September in each experimental year (2023–2025). The station continuously recorded photosynthetic photon flux density (PPFD), which was integrated to obtain daily incident PAR. Because FIPAR was measured on discrete sampling dates rather than continuously, daily values between two adjacent measurements were estimated by linear interpolation. The measurements used for RUE estimation began at late jointing, after canopy closure, thereby excluding the early period of rapid and potentially nonlinear canopy expansion. During these post-closure intervals, FIPAR was near its seasonal maximum and changed relatively gradually; therefore, linear interpolation was considered an appropriate local numerical approximation. This approximation was applied only to FIPAR between adjacent measurement dates and did not assume that leaf area, chlorophyll status, leaf senescence, or other canopy processes changed linearly throughout the growing season. Because FIPAR was determined directly from incident and transmitted PAR, the measured values reflected the integrated effects of canopy characteristics on PAR interception. Similar linear-interpolation procedures have been used to estimate daily FIPAR from periodic field measurements for calculating cumulative intercepted PAR and RUE (Harbo et al., 2022; Mukiibi et al., 2023).

Daily IPAR was calculated as the product of daily incident PAR and interpolated daily FIPAR and was subsequently summed over the corresponding growth interval. For each stage-specific RUE value, aboveground dry-biomass accumulation and cumulative IPAR were determined over the same interval between two adjacent sampling dates. The explicit interpolation formula and detailed calculation procedures are provided in **Supplementary Methods S1**, and the seasonal dynamics of daily and cumulative incident PAR are shown in **Supplementary Figure 2**. Stage-specific RUE was calculated using Equation 2 (Hipps et al., 1983; Kar and Kumar, 2016):

(2) $$RUE=\frac{\Delta B}{\sum IPAR}$$

where Δ*B* is the increase in aboveground biomass per unit ground area during a given growth period (g m^−2^), and ∑*IPAR* is the cumulative intercepted *PAR* over the same period (MJ m^−2^).

#### 2.3.2 Dry matter accumulation, translocation, and partitioning

At the seedling, jointing, silking, grain-filling, and maturity stages, two plants with uniform growth were randomly selected from each experimental plot. Entire plants were carefully excavated using a shovel, and different organs—including stems, leaves, sheaths, tassels, ears, and roots—were separated using scissors and stored individually in labeled paper bags. Samples were first heated at 105 °C for 30 min to terminate enzymatic activity, then dried at 85 °C until nearly constant weight, and finally oven-dried at 75 °C to constant weight for determination of dry matter. Dry matter translocation from vegetative organs (leaves, stems, and leaf sheaths), as well as the pre-silking dry matter translocation of the whole vegetative organs and post-silking dry matter accumulation, were calculated using Equations 3–7 (Ntanos and Koutroubas, 2002; Zhang et al., 2024b):

(3) $$DMT=DM_{S}-DM_{M}$$

(4) $$DMTR=\frac{DMT}{DM_{S}}\times 100$$

(5) $$CDMT=\frac{DMT}{GDM}\times 100$$

(6) DMA=GDM−DMT

(7) $$CDMA=\frac{DMA}{GDM}\times 100$$

Where *DMT* refers to the amount of pre-silking dry matter translocation (g plant^−1^); *DM_S_* and *DM_M_* represent the dry matter at the silking stage and maturity stage, respectively (g plant^−1^); *DMTR* represents the pre-silking dry matter translocation rate (%); *CDMT* represents the contribution of pre-silking dry matter translocation to grain dry matter (%); *GDM* represents the grain dry matter at maturity (g plant^−1^); *DMA* represents the post-silking dry matter accumulation (g plant^−1^); and *CDMA* represents the contribution of post-silking dry matter accumulation to grain dry matter (%). The value of *DMT* was calculated separately for individual vegetative organs (leaf, stem, and sheath) as well as for the combined vegetative organs. In Eq. (7), where *DMA* is calculated, *DMT* specifically refers to the total dry matter translocation from all vegetative organs.

#### 2.3.3 Grain filling characteristics

At the silking stage, representative ears with uniform growth and synchronized silking were selected and tagged in each experimental plot. Beginning 15 days after silking, sampling was conducted at 5–7 d intervals until physiological maturity. At each sampling date, six representative ears were collected from each treatment, consisting of two ears from each of the three replicated plots. From the middle portion of each ear, 100 kernels were sampled and oven-dried at 75 °C to constant weight to determine the 100-kernel dry weight. The grain-filling process was fitted using a logistic growth equation, and the related grain-filling parameters were calculated using Equations 8–13 following Li et al. (2020):

(8) $$y=\frac{a}{\left(1+be^{-kt}\right)}$$

(9) $$T_{\max}=\frac{\ln b}{k}$$

(10) $$W_{\max}=\frac{a}{2}$$

(11) $$R_{\max}=\frac{ak}{4}$$

(12) $$R_{mean}=\frac{0.99ak}{\ln b+4.59512}$$

(13) $$AGP=\frac{6}{k}$$

Where *y* represents the 100-kernel weight at *t* days after silking (g 100 kernels^−1^); *a* represents the theoretical maximum 100-kernel weight (g 100 kernels^−1^); *b* is the initial parameter of the Logistic equation; *k* represents the growth rate parameter; and *t* represents the number of days after silking (d). *T*_max_ represents the time required to reach the maximum grain-filling rate (d); *W*_max_ represents the 100-kernel weight at the maximum grain-filling rate (g 100 kernels^−1^); *R*_max_ and *R*_mean_ represent the maximum and mean grain-filling rates, respectively (g 100 kernels^−1^ d^−1^); and *AGP* represents the active grain-filling period (d). In this study, kernel dry-weight accumulation and the derived parameters *W*_max_, *R*_max_, *R*_mean_, *T*_max_, and *AGP* were collectively referred to as grain-filling characteristics.

#### 2.3.4 Yield

At physiological maturity, 25 ears were randomly harvested from each plot to determine yield components and grain yield. Grain weight was measured and then adjusted to a standard moisture content of 14%, after which grain yield was converted to a per-hectare basis (kg ha^−1^) according to the planting density.

### 2.4 Data analysis

Statistical analyses were performed using SPSS Statistics 27.0 (IBM Corporation, Armonk, NY, USA). A two-way analysis of variance (Two-way ANOVA) was conducted to evaluate the effects of planting density (D), fertilization treatment (F), and their interaction (D × F) on dry matter accumulation, translocation parameters, and yield-related traits. Type III sums of squares were used to assess the significance of each factor. Mean comparisons among treatments were performed using Tukey’s honestly significant difference (HSD) test at P< 0.05. Pearson correlation analysis was conducted to examine relationships among the measured variables. Mantel tests were performed using the “vegan” package in R (version 4.4.2; R Core Team, Vienna, Austria), and correlation matrices were visualized using the “corrplot” package. Random forest regression was used to assess the relative importance of agronomic and physiological traits in explaining maize yield, with variable importance quantified by the percentage increase in mean squared error (%IncMSE). Model performance was evaluated using out-of-bag (OOB) prediction. To further explore the direct and indirect effects of planting density and fertilization on dry matter translocation and grain yield, a partial least squares path model (PLS-PM) was constructed using the “plspm” package in R. Model performance was evaluated using the goodness-of-fit (GoF) index. All figures were generated using Origin 2024b (OriginLab Corporation, Northampton, MA, USA) and R software.

## 3 Results

### 3.1 FIPAR

**Figure 2:**
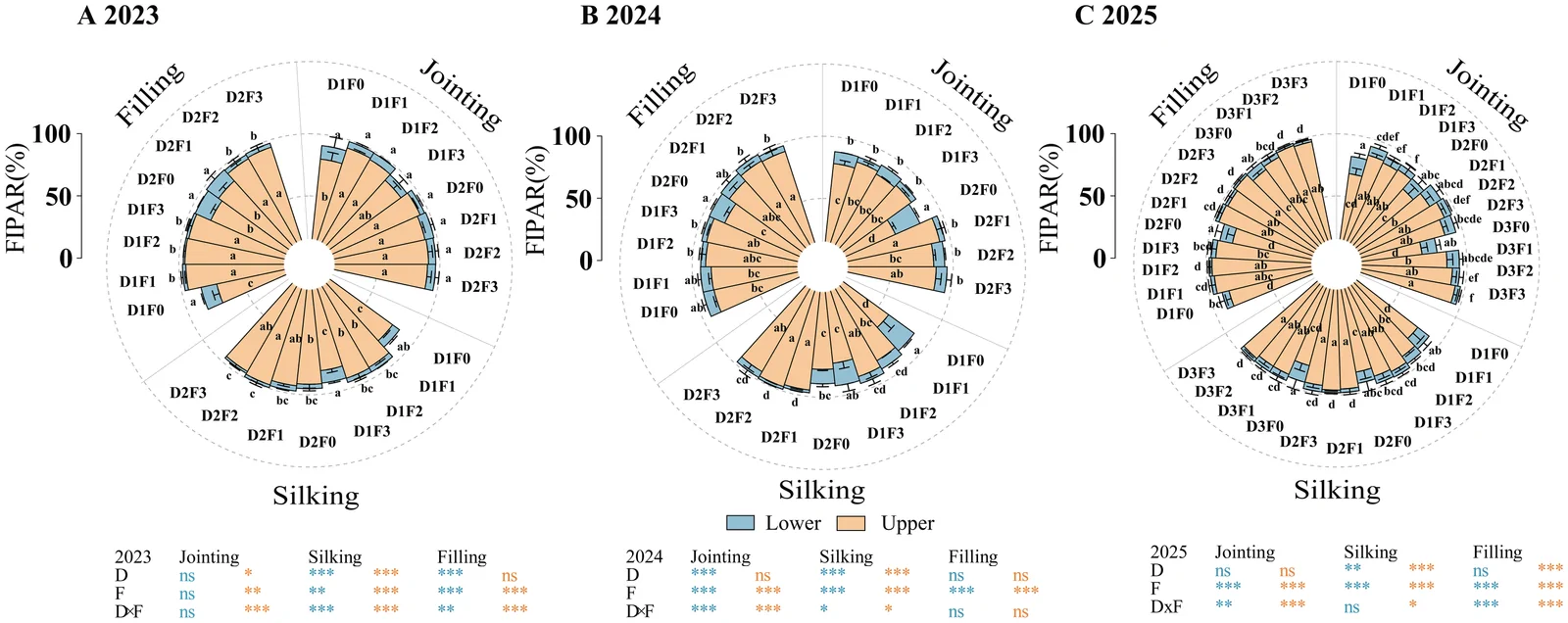
Effects of different planting densities and fertilization treatments on the fraction of intercepted photosynthetically active radiation (FIPAR) at the jointing **(A)**, silking **(B)**, and grain-filling **(C)** stages of maize from 2023 to 2025. D1, D2, and D3 represent planting densities of 70,000, 100,000, and 130,000 plants ha^−1^, respectively; F0, F1, F2, and F3 represent no fertilization, chemical fertilizer only, 17% and 34% organic fertilizer substitution, respectively. D indicates the effect of planting density, F indicates the effect of fertilization, and D×F indicates the interaction effect between planting density and fertilization. Significance levels: *P< 0.05, **P< 0.01, ***P< 0.001; ns, not significant.

Total canopy FIPAR increased progressively as the growing period advanced, concomitant with an intensifying vertical heterogeneity in light distribution. This was characterized by increased interception in the upper canopy and a corresponding decline in the lower layers. Across all treatments and key growth stages, the upper canopy consistently accounted for more than 60% of the total intercepted radiation. Planting density exerted a significant effect on FIPAR (**Figure 2**). Compared with the D1 treatment, D2 significantly augmented total FIPAR across all growth stages, with increments ranging from 0.99% to 6.25%. However, further increasing the density to D3 in 2025 did not yield any additional significant gains. Similarly, fertilization significantly enhanced FIPAR; F2 increased FIPAR by 6.55%–8.28% relative to F0 but did not differ significantly from F1.

### 3.2 Stage-specific RUE

**Figure 3:**
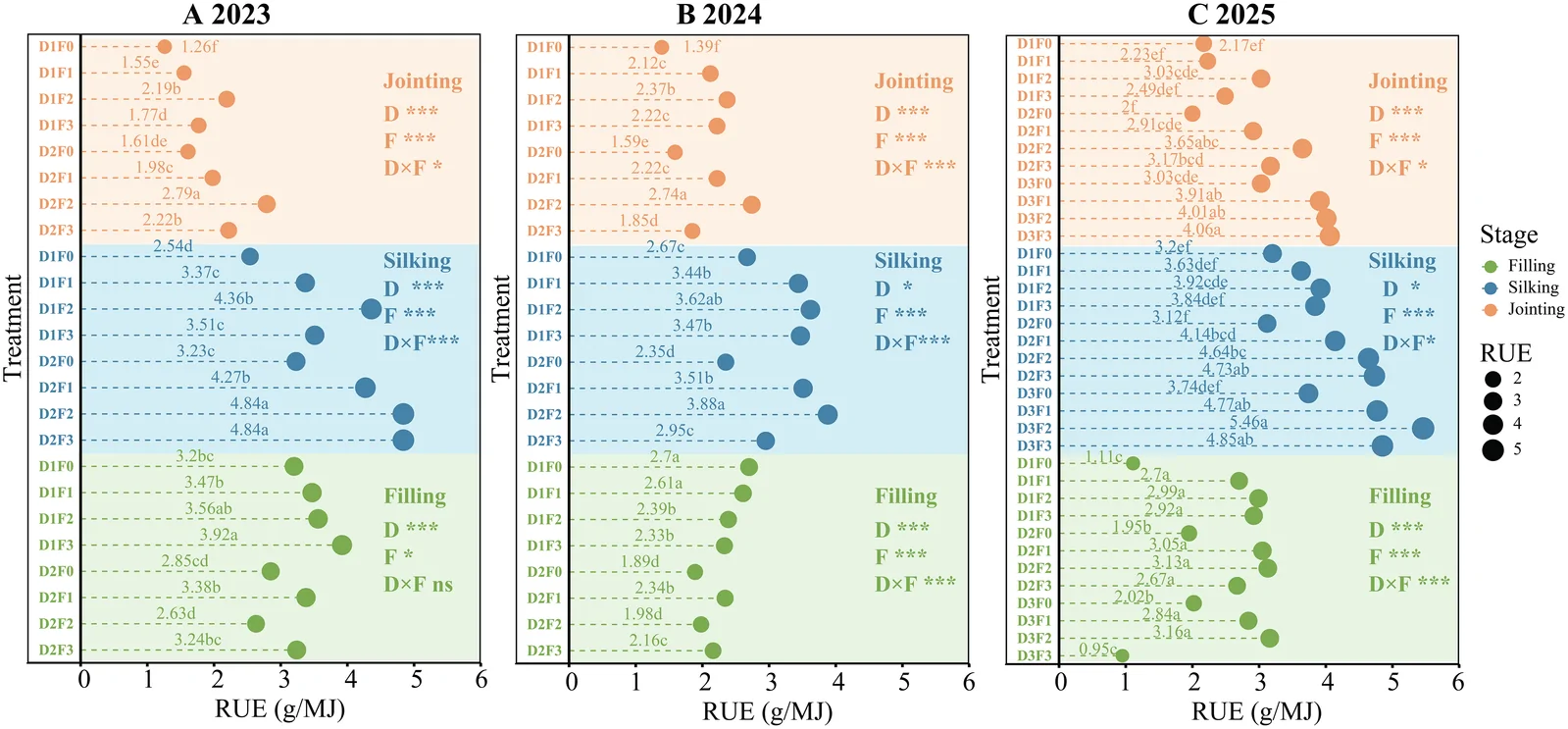
Effects of different planting densities and fertilization treatments on radiation use efficiency (RUE) at the jointing **(A)**, silking **(B)**, and grain-filling **(C)** stages of maize from 2023 to 2025. D1, D2, and D3 represent planting densities of 70,000, 100,000, and 130,000 plants ha^−1^, respectively; F0, F1, F2, and F3 represent no fertilization, chemical fertilizer only, 17% and 34% organic fertilizer substitution, respectively. D indicates the effect of planting density, F indicates the effect of fertilization, and D×F indicates the interaction effect between planting density and fertilization. Significance levels: *P< 0.05, **P< 0.01, ***P< 0.001; ns, not significant.

Stage-specific RUE exhibited a unimodal trend over the growing period, peaking at the silking stage(**Figure 3**). Planting density, fertilization treatment, and their interaction significantly influenced RUE (*P*< 0.05). Specifically, increasing planting density significantly enhanced RUE; compared with D1, D2 increased RUE at the silking stage by 24.67%, 3.70%, and 13.98% in 2023, 2024, and 2025, respectively (*P*< 0.05). The D3 treatment in 2025 further improved RUE, exceeding D1 and D2 by 29.04% and 13.17%, respectively. Fertilization consistently elevated RUE, with F2 exhibiting a superior performance over other regimes. At the silking stage, RUE under F2 was 7.76%–20.42% higher than under F1. Among all combinations, D2F2 achieved the highest RUE in 2023 and 2024, surpassing D1F1 by 12.79%–43.62%. In 2025, the maximum RUE was recorded under the D3F2 treatment, reaching 5.46 g MJ^−1^.

### 3.3 Dry matter accumulation and partitioning

**Figure 4:**
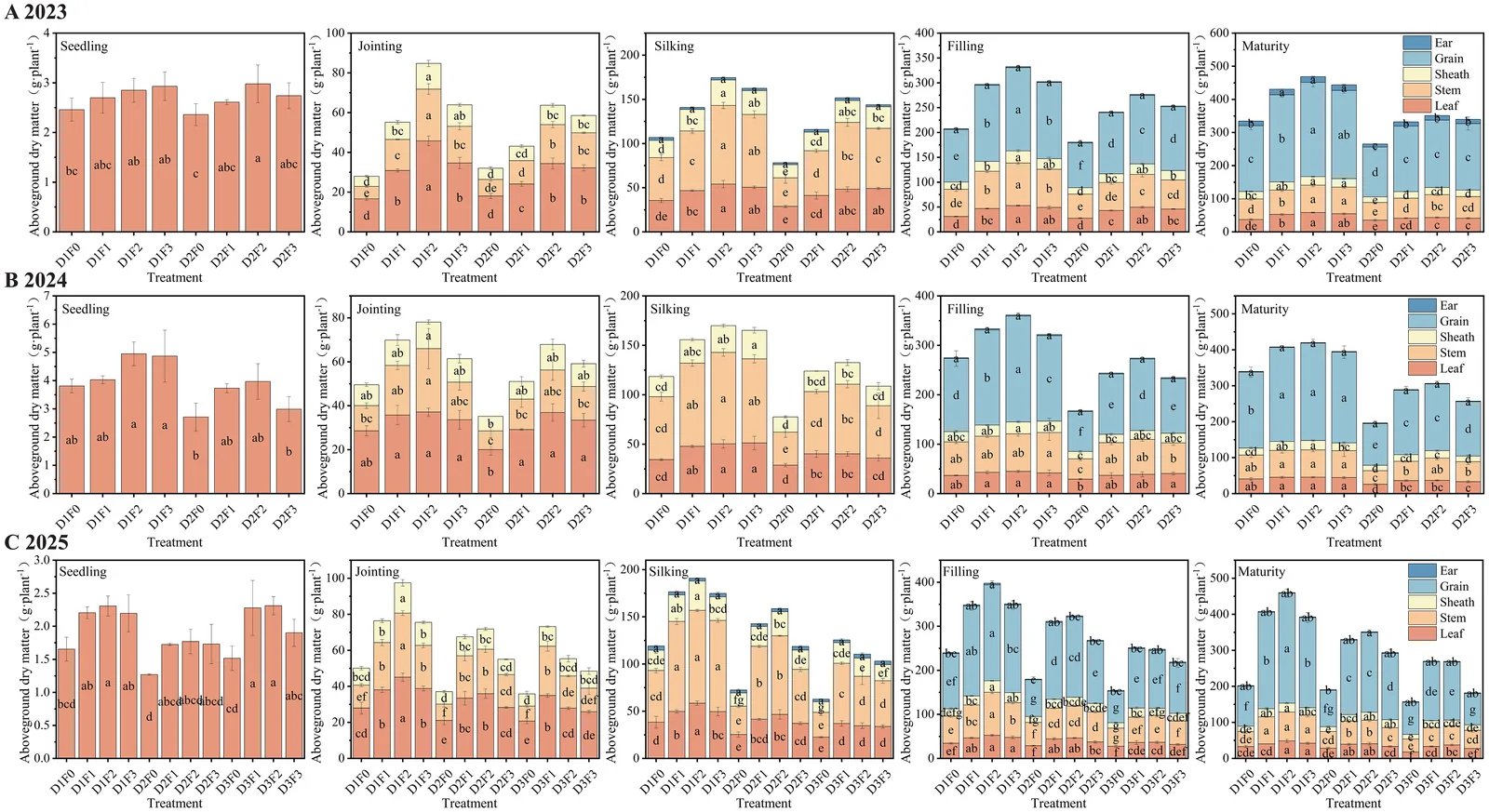
Effects of planting density and fertilization treatment on aboveground dry matter accumulation and partitioning per plant (g plant^−1^) at different growth stages of maize in 2023 **(A)**, 2024 **(B)**, and 2025 **(C)**. Growth stages include seeding, jointing, silking, filling, and maturity. Different colors represent dry matter of different organs: Ear, Grain, Sheath, Stem, and Leaf. D1, D2, and D3 represent planting densities of 70,000, 100,000, and 130,000 plants ha^−1^, respectively; F0, F1, F2, and F3 represent no fertilization, chemical fertilizer only, 17% and 34% organic fertilizer substitution, respectively. Different lowercase letters within bars indicate significant differences among treatments at the same growth stage (*P*< 0.05). Error bars indicate standard errors.

Aboveground dry matter accumulation per plant increased continuously throughout the growing period. Increasing planting density significantly reduced per-plant aboveground dry matter accumulation. At maturity, aboveground dry matter accumulation per plant under D2 was 20.29%–34.80% lower than that under D1, while D3 reduced it by 36.53% relative to D1 in 2025 (**Figure 4**). Fertilization slightly enhanced aboveground dry matter accumulation per plant. Compared with F1, F2 increased per-plant aboveground dry matter accumulation by 0.30%, 1.93%, and 6.28% in 2023, 2024, and 2025, respectively. Regarding dry matter partitioning, the proportion of dry matter allocated to leaves gradually decreased with crop development, whereas the proportion allocated to stems increased initially and then declined. In contrast, the proportion of dry matter allocated to grain increased from the grain-filling stage and reached the maximum at maturity (**Supplementary Figure 3**). With increasing planting density, the proportion of grain dry matter gradually decreased. In contrast, the combined application of organic and inorganic fertilizers significantly increased the proportion of stem dry matter at the silking stage, and under the D1 treatment the grain dry matter proportion at maturity under F2 was 1.90%–5.44% higher than that under F1.

### 3.4 Dry matter translocation and contribution to grain yield

The DMT of individual vegetative organs followed the general order: stem > leaf blade > leaf sheath (**Table 1**). Compared with D1, D2 significantly increased DMT from both stems and leaf blades; however, DMT per plant declined markedly under D3, with stem DMT under the F3 treatment decreasing by more than 70% in 2025. The stem DMT under the D2F2 treatment was 55.98%–178.98% higher than that under D1F1.

**Table 1:** Effects of planting density and fertilization on pre-silking dry matter translocation from different organs and post-silking dry matter contribution to grain dry matter of maize from 2023 to 2025.

Year	Density	Fertilizer	Leaf			Stem			Sheath			CDMA (%)
			DMT (g plant^-1^)	DMTR (%)	CDMT (%)	DMT (g plant^-1^)	DMTR (%)	CDMT (%)	DMT (g plant^-1^)	DMTR (%)	CDMT (%)	
2023	D1	F0	5.46abc	12.48ab	2.81ab	3.86d	5.9e	1.96e	2.37a	9.48a	1.19a	93.8a
		F1	1.49c	2.62b	0.56b	13.63bc	15.56bcd	5.16cd	1.73a	6.57a	0.66a	93.22ab
		F2	0.94c	1.58b	0.34b	22.46a	21.44ab	7.92b	2.87a	9.95a	1.03a	90.64abc
		F3	1c	1.8b	0.38b	9.28cd	10.31de	3.48de	1.52a	5.58a	0.57a	95.43a
	D2	F0	4.6bc	11.5ab	3.07ab	9.26cd	14.79cd	6.17bc	0.73a	4.04a	0.49a	90.08abc
		F1	5.18abc	11.05ab	2.62ab	16.59ab	21.56ab	8.38ab	1.33a	6.21a	0.67a	88.13bc
		F2	12.2a	21.7a	5.96a	21.26a	23.53a	10.45a	3.77a	14.66a	1.83a	81.47d
		F3	9.33ab	18.2a	4.6a	13.43bc	16.98bc	6.67bc	1.83a	8.43a	0.89a	87.59c
ANOVA	D		***	***	**	***	***	ns	ns	ns	***	***
	F		ns	Ns	***	***	***	ns	ns	ns	***	***
	D×F		**	**	ns	ns	ns	ns	ns	ns	ns	ns
2024	D1	F0	0.98b	2.38b	0.48b	4.79c	6.4c	2.33c	4.64cd	19.18bc	2.22bc	94.62a
		F1	4.74ab	9.19ab	1.8ab	16.81ab	18.42ab	6.43ab	12.13ab	32.58ab	4.63abc	86.81bc
		F2	8.24a	15.15a	3.07ab	22.92a	23.26a	8.47a	14.42a	35.27ab	5.31ab	83.04c
		F3	6.53ab	12.6ab	2.57ab	10.82bc	12.69bc	4.28bc	13.6ab	38.59a	5.39ab	87.79bc
	D2	F0	0.51b	1.92b	0.44b	10.09bc	20.98ab	8.64a	1.86d	10.46c	1.56c	89.54ab
		F1	4.82ab	11.3ab	2.68ab	11.53bc	17.54ab	6.4ab	12.69ab	40.27a	7.1a	83.93c
		F2	7.93a	17.42a	4.25a	10.49bc	14.4abc	5.66abc	8.54abc	29.58ab	4.59abc	85.32bc
		F3	4.49ab	11.73ab	2.88ab	10.83bc	17.57ab	6.97ab	8.02bc	31.4ab	5.13ab	84.91bc
ANOVA	D		ns	Ns	**	ns	*	**	ns	ns	***	*
	F		***	***	***	ns	ns	***	***	***	***	***
	D×F		ns	Ns	***	***	***	ns	ns	ns	**	*
2025	D1	F0	4.15bc	11.76b	3.74c	21.06bc	33.69ab	19.07a	5.31a	26.19a	4.82ab	72.2cd
		F1	7.88abc	16.27ab	2.93c	7.47de	8.99ef	2.78fg	5.34a	19.13a	2bc	92.33a
		F2	7.8abc	13.55ab	2.6c	20.78bc	20.61bcde	6.81defg	5.28a	17.45a	1.74c	88.46ab
		F3	3.47bc	7.24b	1.32c	9.59de	10.89def	3.91efg	7.37a	26.23a	2.93bc	91.64a
	D2	F0	1.7c	5.85b	1.69c	0.96e	2.01f	0.94g	4.58a	25.87a	4.53abc	92.74a
		F1	6.32bc	13.82ab	3.06c	13.9cd	17.65cde	6.68defg	5.53a	22.61a	2.67bc	87.58ab
		F2	6.55abc	13.81ab	2.96c	20.84bc	24.16bcd	9.41cdef	3.69a	14.36a	1.67c	85.98ab
		F3	15.68a	31.81a	8.54b	33.33a	39.07a	18.14ab	3.4a	13.13a	1.81c	71.29d
	D3	F0	8.29abc	31.84a	9.3b	9.1de	20.05bcde	10.2cdef	4.01a	24.07a	4.49bc	75.85cd
		F1	9.27abc	21.79ab	5.68bc	26.06ab	33.07ab	15.98abc	4.91a	20.67a	3.03bc	74.95cd
		F2	3.3bc	8.07b	2.04c	20.86bc	28.25abc	12.94abcd	6.43a	28.14a	4bc	80.74bc
		F3	11.99ab	30.18a	13.89a	9.62de	15.88cdef	11.06bcde	6.43a	29.59a	7.44a	68.25d
ANOVA	D		**	***	ns	*	***	ns	ns	***	ns	***
	F		**	***	***	ns	ns	ns	ns	***	***	***
	D×F		***	***	***	***	***	ns	ns	***	***	***

DMT, pre-silking dry matter translocation; DMTR, pre-silking dry matter translocation rate; CDMT, contribution of pre-silking dry matter to grain; CDMA, contribution of post-silking dry matter to grain. D1, D2, and D3 represent planting densities of 70,000, 100,000, and 130,000 plants ha^−1^, respectively; F0, F1, F2, and F3 represent no fertilization, chemical fertilizer only, 17% and 34% organic fertilizer substitution, respectively. D, F, and D×F indicate the effects of planting density, fertilization, and their interaction, respectively. Different lowercase letters within the same year indicate significant differences among treatments (*P*< 0.05); *P< 0.05, **P< 0.01, ***P< 0.001; ns, not significant.

**Figure 5:**
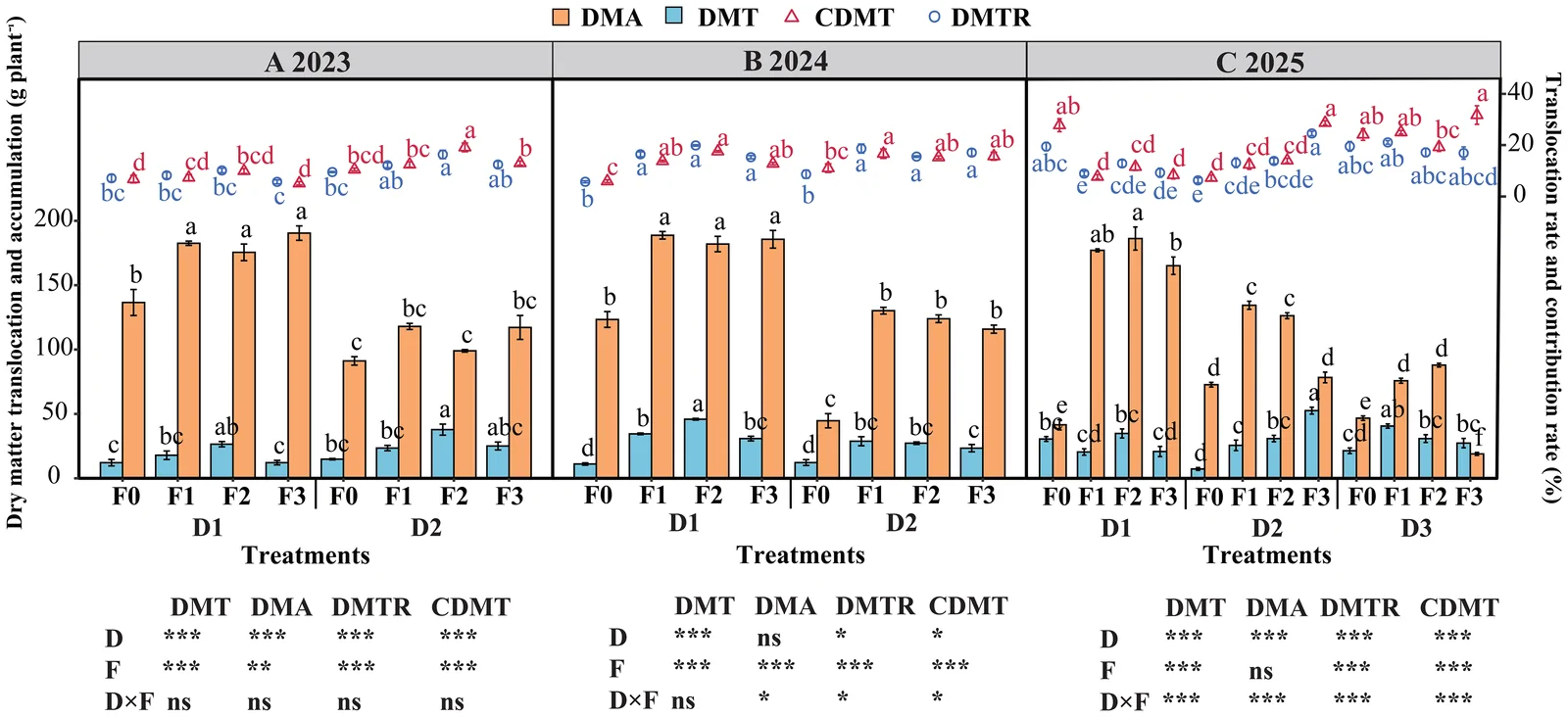
Effects of planting density and fertilization on pre-silking dry matter translocation (DMT), post-silking dry matter accumulation (DMA), translocation rate (DMTR), and contribution to grain (CDMT) of maize in 2023 **(a)**, 2024 **(b)**, and 2025 **(c)**. D1, D2, and D3 represent planting densities of 70,000, 100,000, and 130,000 plants ha^−1^, respectively; F0, F1, F2, and F3 represent no fertilization, chemical fertilizer only, 17% and 34% organic fertilizer substitution, respectively. D, F, and D×F indicate the effects of planting density, fertilization, and their interaction, respectively. Different lowercase letters indicate significant differences among treatments (*P*< 0.05); *P< 0.05, **P< 0.01, ***P< 0.001; ns, not significant. Error bars indicate standard errors.

Planting density and fertilization treatment significantly affected DMT, DMTR and CDMT (**Figure 5**; **Table 1**). DMT, DMTR, and CDMT all increased with rising planting density. Relative to D1, CDMT under D2 was higher by 95.95%, 17.93%, and 12.71% in 2023, 2024, and 2025, respectively. The DMT under F2 was on average 11.37%–55.35% greater than that under F1. In contrast, increasing planting density significantly reduced per plant DMA and CDMA. Compared with D1, DMA and CDMA under D2 decreased by 27.76%–37.91% and 2.04%–6.93%, respectively. Across all treatments, CDMA consistently exceeded 80%, confirming that post-silking assimilation remained the predominant source of grain dry matter per plant.

### 3.5 Grain filling characteristics

**Figure 6:**
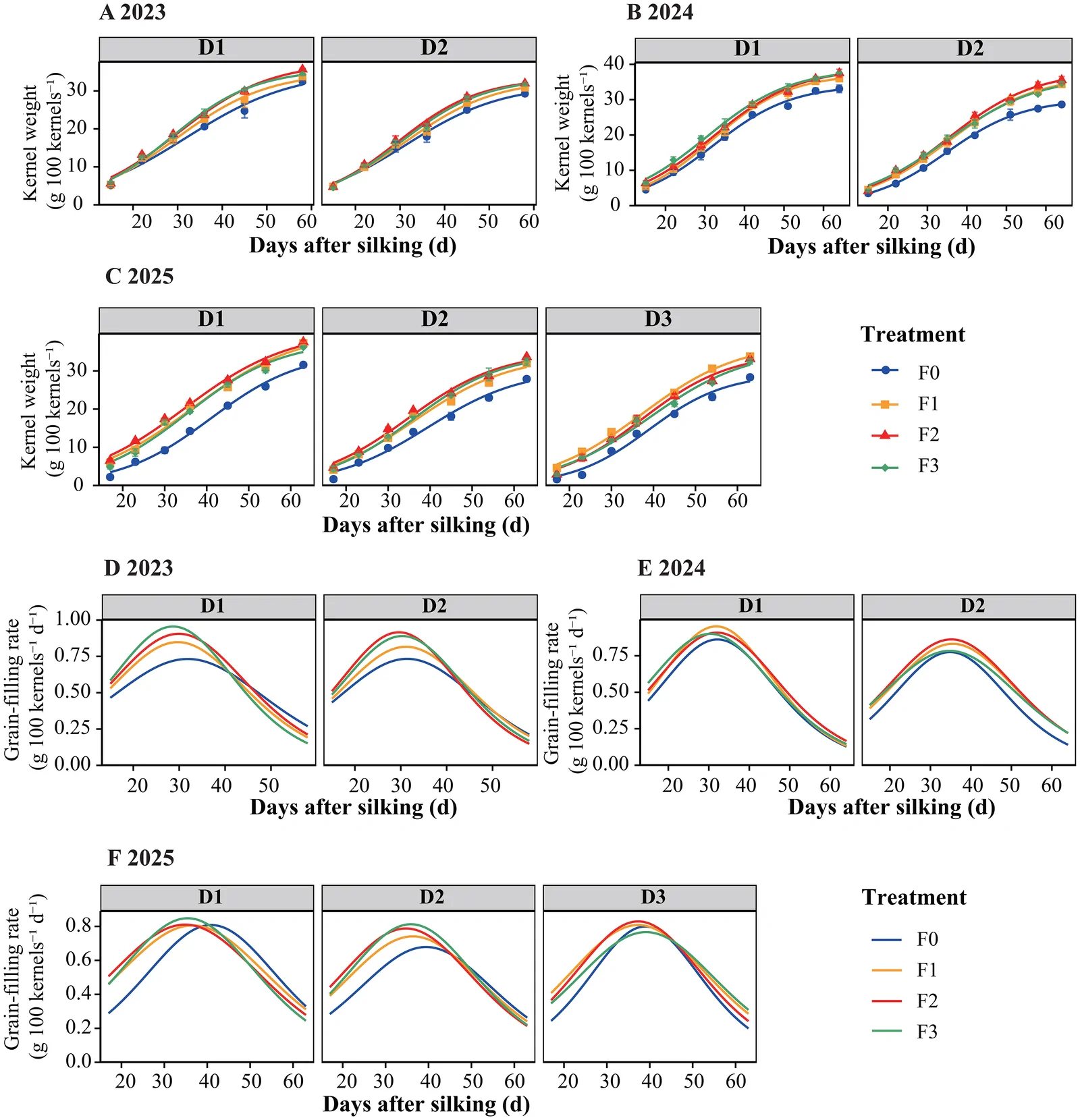
Effects of planting density and fertilization on grain-filling dynamics fitting **(A–C)** and grain-filling rate after silking **(D–F)** of maize from 2023 to 2025. D1, D2, and D3 represent planting densities of 70,000, 100,000, and 130,000 plants ha−1, respectively; F0, F1, F2, and F3 represent no fertilization, chemical fertilizer only, and 17% and 34% organic fertilizer substitution, respectively. D, F, and D×F indicate the effects of planting density, fertilization, and their interaction, respectively.

Kernel dry weight accumulation followed a typical sigmoidal growth pattern, commencing at 15–17 days after silking (DAS) and reaching physiological maturity at 58–63 DAS. The Logistic equation provided an excellent fit to the kernel dry weight accumulation curves across all treatments from 2023 to 2025 (R^2^ = 0.9789–0.9994). Both planting density and fertilization treatment exerted significant effects on grain-filling characteristics of spring maize (**Figures 6A–C**; **Supplementary Table 2**). Kernel dry weight decreased significantly with increasing planting density, consistently following the order D1 > D2 > D3. Fertilization treatments significantly enhanced kernel dry weight relative to the unfertilized control; compared with F0, the mean kernel dry weight under F1, F2, and F3 increased by 12.48% (*P*< 0.05), 14.36% (*P*< 0.01), and 11.90% (*P*< 0.01), respectively.

Grain-filling parameters varied significantly among treatments among (**Figures 6D–F**; **Supplementary Table 2**). R_max_ ranged from 0.679 to 0.956 g 100-kernel^−1^ d^−1^, with the highest value observed under D1F3 in 2023 and the lowest under D2F0 in 2025. Across treatments, R_mean_ was 0.432 ± 0.044 g 100-kernel^−1^ d^−1^, and D1 was significantly higher than D2 and D3 by 10.15% and 15.29%, respectively (*P*< 0.01). Under D2, R_max_ and R_mean_ were 3.61%–12.20% and 2.33%–9.30% higher with F2 than with F1, respectively. Increasing planting density increased T_max_, with D2 exhibiting a delay of 3.52 d relative to D1. AGP ranged from 54.68 to 75.28 d. In 2025, AGP was 3.90 and 6.34 d shorter under D2 and D3, respectively, than under D1, with the shortest AGP observed under D3F0.

### 3.6 Grain yield

Planting density and fertilization treatments both had highly significant effects on maize yield (*P* ≤ 0.01), whereas their interaction was significant only in some years (**Table 2**). Grain yield generally increased from D1 to D2, whereas a further increase to D3 reduced yield in 2025. The D2 treatment consistently produced the highest yield over the three years, increasing yield by 5.03%–25.73% (2023), 1.30%–6.71% (2024), and 9.27%–11.74% (2025) compared with D1. When density further increased to D3, yield decreased significantly by 8.86%–13.37% relative to D2 in 2025. The yield improvement under higher density was mainly attributed to an increase in ear number per unit area; however, this was accompanied by reductions in 1000-kernel weight (8.15%–11.29%) and kernels per ear (16.85%–17.00%), as well as an increase in barren tip length (22.88%–59.39%). Fertilization significantly increased grain yield. Compared with F0, yields under F1, F2, and F3 increased by 42.39%, 49.15%, and 38.51%, respectively. Among all treatments, D2F2 achieved the highest yield over the three years, reaching 18.58, 19.01, and 19.23 t ha^−1^, respectively, representing a 7.46%–23.82% increase compared with D1F1.

**Table 2:** Effects of planting density and fertilization on yield and yield components of maize from 2023 to 2025.

Year	Density	Fertilizer	1000-kernelweight (g)	Ear length (cm)	Ear diameter (cm)	Kernels per row	kernels per ear	Bare tip length (cm)	Yield (t ha^-1^)
2023	D1	F0	284.38e	20.40c	52.16b	36.89b	650.86b	1.61b	13.72e
		F1	336.04b	21.56a	53.57a	41.15a	678.15a	1.26de	16.76c
		F2	356.52a	21.71a	53.64a	40.67a	676.46ab	0.81g	17.69bc
		F3	338.18b	21.03b	53.68a	40.69a	665.56ab	1.04f	15.77d
	D2	F0	272.36f	17.88f	49.90e	32.50d	533.23e	1.81a	17.25c
		F1	310.77d	19.44d	51.42cd	34.92c	546.91de	1.36cd	18.89a
		F2	318.37c	19.53d	50.89d	35.20c	578.14c	1.08ef	18.58ab
		F3	306.50d	18.50e	51.59c	32.69d	562.73cd	1.55bc	16.90c
ANOVA	D		***	***	***	***	***	***	***
	F		***	***	***	***	*	***	***
	D×F		***	ns	ns	*	ns	ns	***
2024	D1	F0	329.95c	178.50c	51.26d	35.00c	630.00b	1.01b	13.42c
		F1	369.55a	199.58a	54.19a	38.89b	716.89a	0.77cd	17.69ab
		F2	371.93a	198.07a	53.38b	40.30a	698.44a	0.77cd	18.20ab
		F3	363.13a	199.50a	53.34b	39.25ab	716.00a	0.75d	17.04b
	D2	F0	292.56d	150.11d	47.78e	30.40d	523.00d	1.65a	14.32c
		F1	346.58b	190.50b	51.84cd	35.14c	571.43c	1.43ab	17.92ab
		F2	345.15b	179.57c	52.01c	35.20c	597.20c	1.23b	19.01a
		F3	343.34b	178.27c	51.50cd	34.50c	600.20c	1.10bc	17.52ab
ANOVA	D		***	***	***	***	***	***	*
	F		***	***	***	***	***	**	***
	D×F		ns	***	**	ns	ns	ns	ns
2025	D1	F0	315.80e	20.98b	49.45ef	38.86cd	595.00c	2.01c	11.02d
		F1	372.72ab	21.94a	53.83a	40.71a	696.57a	1.02fgh	15.53c
		F2	376.05a	21.34ab	53.09a	38.33bc	664.33b	0.80h	17.29abc
		F3	362.27b	21.22b	52.26b	39.79ab	704.57a	0.93gh	16.27bc
	D2	F0	280.66f	16.55e	45.02h	24.64g	378.86e	3.46a	8.38e
		F1	320.54e	19.65cd	48.09g	35.00ef	553.27d	1.47de	16.97abc
		F2	332.84cd	19.06de	50.73c	34.33f	601.00c	1.14efgh	19.23a
		F3	331.65cd	19.45d	50.38cd	36.43de	603.29c	1.13efgh	18.18ab
	D3	F0	282.60f	16.54e	45.55h	23.40g	365.41e	2.95b	7.26e
		F1	337.70c	20.06c	49.98cde	36.31de	580.15cd	1.51d	17.80abc
		F2	331.39cd	19.19de	49.61def	35.08ef	578.00cd	1.29def	17.33abc
		F3	325.09de	18.80e	48.99f	34.42f	556.50d	1.24defg	16.57bc
ANOVA	D		***	***	***	***	***	***	*
	F		***	***	***	***	***	***	***
	D×F		ns	***	***	***	***	**	***

D1, D2, and D3 represent planting densities of 70,000, 100,000, and 130,000 plants ha^−1^, respectively; F0, F1, F2, and F3 represent no fertilization, chemical fertilizer only, 17% and 34% organic fertilizer substitution, respectively. D, F, and D×F indicate the effects of planting density, fertilization, and their interaction, respectively. Different lowercase letters within the same year indicate significant differences among treatments (*P*< 0.05); *P< 0.05, **P< 0.01, ***P< 0.001; ns, not significant.

### 3.7 Correlation analysis and partial least squares path modeling

**Figure 7:**
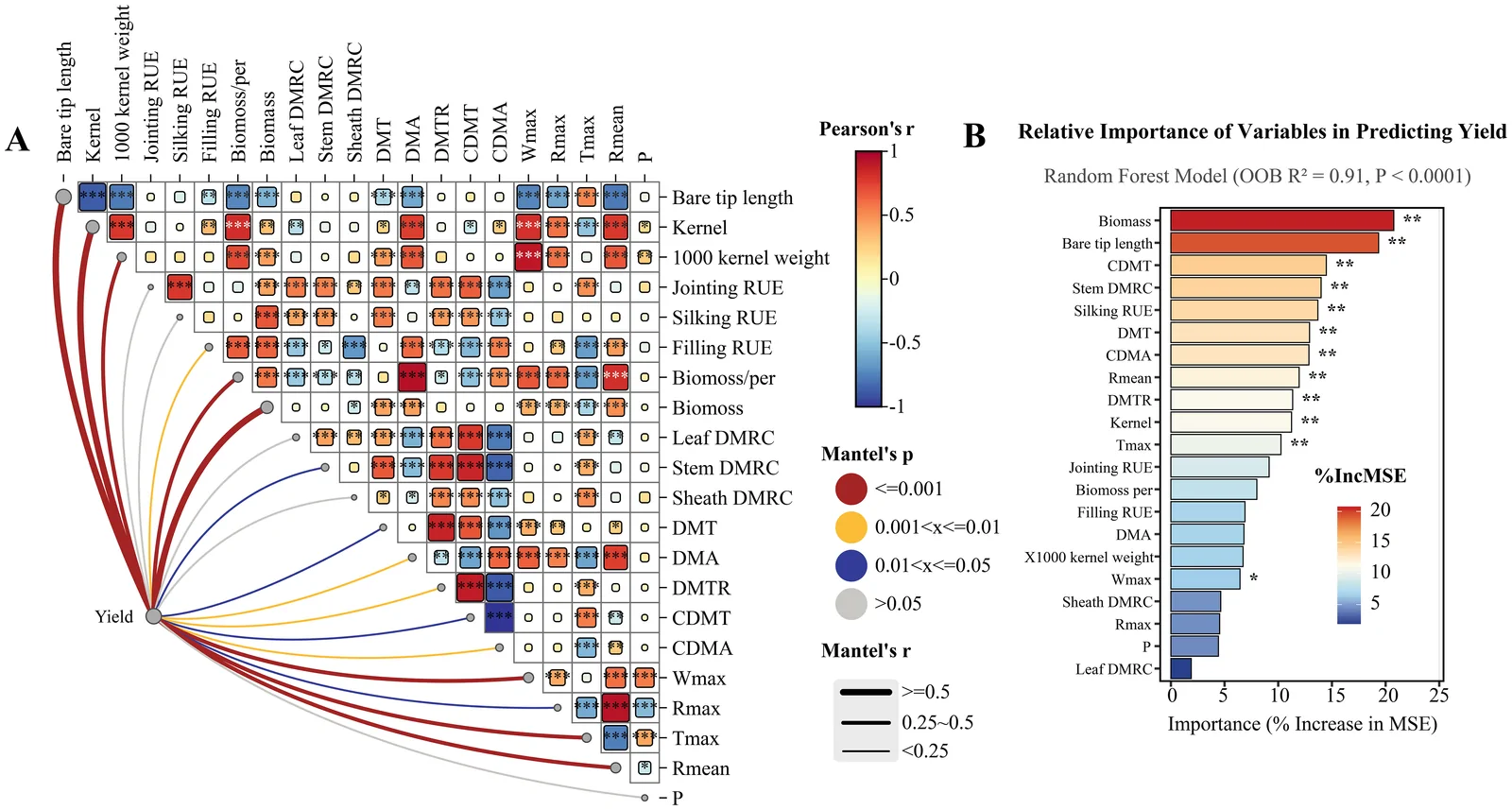
Relationships among agronomic traits and their relative importance in predicting maize grain yield. **(A)** Pearson correlation and Mantel test analysis between maize grain yield and related agronomic traits. The triangular heatmap shows Pearson correlation coefficients among traits; red and blue colors indicate positive and negative correlations, respectively. Asterisks indicate significance levels for Pearson correlations: *P< 0.05, **P< 0.01, and ***P< 0.001. Curved lines indicate Mantel test associations between grain yield and individual traits. Line color represents Mantel significance levels, and line width represents the strength of Mantel’s (r) **(B)** Random forest analysis showing the relative importance of different traits in predicting maize grain yield. Variable importance was quantified as the percentage increase in mean squared error (%IncMSE), with higher values indicating greater contribution to yield prediction. Asterisks indicate significance levels of variable importance based on permutation tests: *P< 0.05, **P< 0.01, and ***P< 0.001. RUE: radiation use efficiency; DMT: pre-silking dry matter translocation; DMA: post-silking dry matter accumulation; DMTR: pre-silking dry matter translocation rate; CDMT: contribution of pre-silking dry matter to grain dry matter; CDMA: contribution of post-silking dry matter to grain dry matter; W_max_: maximum grain weight; Rmax: maximum grain filling rate; T_max_: time to maximum grain filling rate; R_mean_: mean grain filling rate; AGP: active grain-filling period.

The Mantel test indicated that grain yield was highly significantly correlated with aboveground biomass, barren tip length, kernels per ear, 1000-kernel weight, and radiation use efficiency during the grain-filling stage (*P* ≤ 0.001) (**Figure 7A**). Pearson correlation analysis further revealed that yield-related traits were significantly and positively associated with biomass accumulation, radiation use efficiency at different growth stages, and dry matter translocation parameters. In contrast, barren tip length exhibited negative correlations with yield components. To further explore the primary drivers of yield, this study developed a random forest regression model (**Figure 7B**). The results indicated that the model exhibited high predictive and explanatory capacity for grain yield (OOB *R*^2^ = 0.91, *P*< 0.0001). Variable importance analysis identified aboveground biomass as the most important predictor of yield (%IncMSE > 20%) and showed that R_max_ and R_mean_ contributed more strongly to yield prediction than AGP.

**Figure 8:**
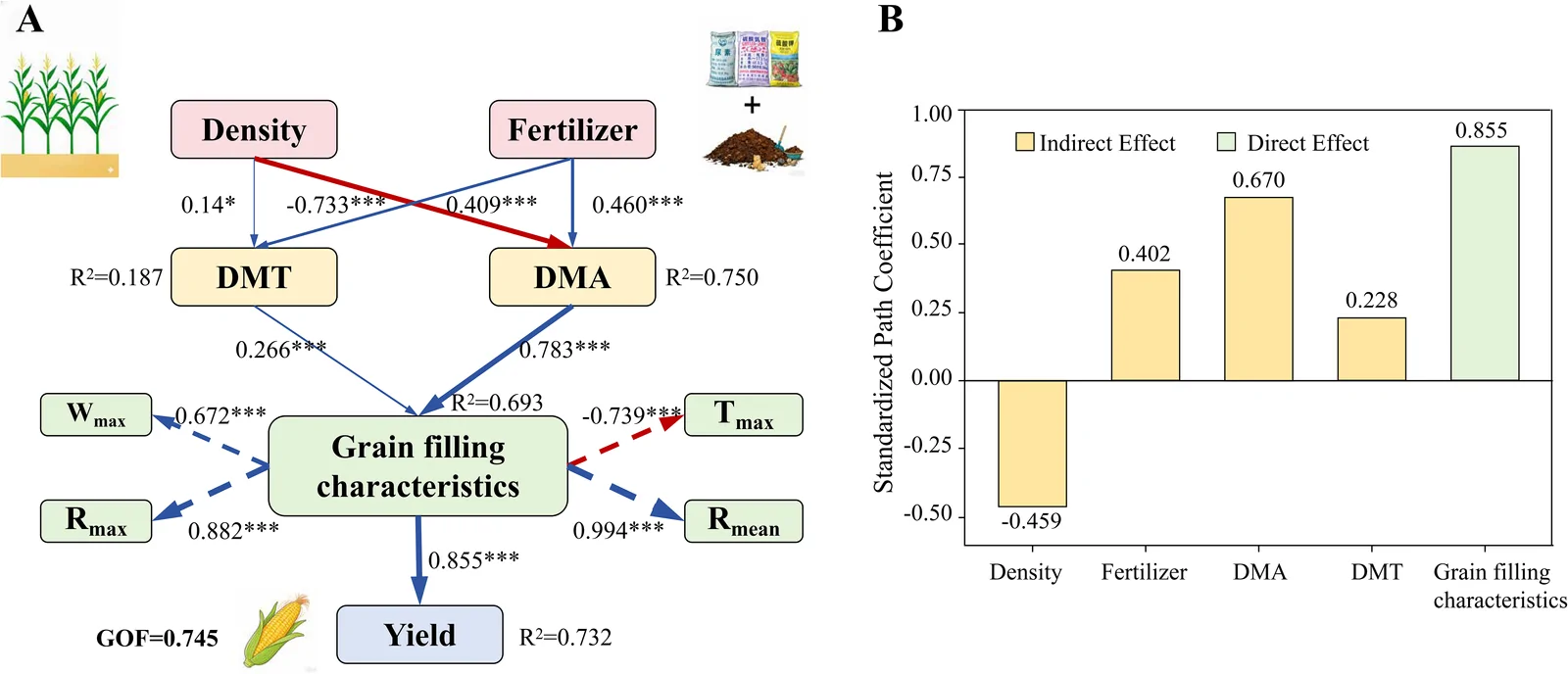
Partial least squares path modeling (PLS-PM) analysis of regulatory pathways of planting density and fertilization on grain -filling characteristics and yield of maize. **(A)** Path model describing causal relationships among planting density, fertilization level, dry matter accumulation and translocation, grain filling characteristics, and yield. Grain filling characteristics serve as a latent variable represented by W_max_, R_max_, T_max_, and R_mean_. Blue and red solid arrows indicate significant positive and negative paths (*P*< 0.05), respectively; gray dashed arrows indicate non-significant paths. Values and line widths on arrows represent standardized path coefficients; R^2^ values indicate variance explained for endogenous variables. **(B)** Standardized total effects of each predictor on yield, including direct and indirect effects. *P< 0.05, ***P< 0.001. DMT: pre-silking dry matter translocation; DMA: post-silking dry matter accumulation; W_max_: maximum grain weight; R_max_: maximum grain filling rate; T_max_: time to maximum grain filling rate; R_mean_: mean grain filling rate; GOF: goodness of fit index.

At the individual-plant scale, the PLS-PM analysis revealed multiple pathways by which planting density and fertilization influence maize yield through DMT, DMA, and grain-filling characteristics (**Figure 8A**). The model showed strong overall fit (GOF = 0.745) and accounted for 73.2% of the variation in grain yield. The results showed that fertilization positively regulated the process of dry matter formation, significantly enhancing DMT (λ = 0.409, *P*< 0.001) and DMA (λ = 0.460, *P*< 0.001). Conversely, planting density exhibited a slight positive influence on DMT (λ = 0.14, *P*< 0.05) but significantly suppressed DMA (λ = −0.733, *P*< 0.001). Both path and total effect analyses demonstrated that high planting density significantly reduced yield primarily by restricting post-silking DMA, with a standardized effect of −0.459. In contrast, fertilization enhanced dry matter accumulation and translocation and improved the grain-filling process (λ = 0.783, *P*< 0.001), resulting in a significant positive effect on yield formation (standardized effect = 0.413) and partly mitigating the source limitation imposed by high planting density (**Figure 8B**).

## 4 Discussion

### 4.1 Effects of planting density and organic fertilizer substitution on canopy radiation characteristics

FIPAR and its conversion efficiency into chemical energy form the basis of crop yield determination. In the context of growing global food security challenges, increasing planting density has become a key strategy for enhancing crop yield potential by forming populations with high light-use efficiency (Tian et al., 2022a; Sun et al., 2023). Our results showed that increasing planting density from D1 to D2 enhanced canopy FIPAR by 0.99%–6.25% (**Figure 2**), consistent with Zhai et al. (2022), who reported that a moderate increase in planting density improves canopy radiation interception by increasing leaf area index and reducing radiation transmission losses (Monteith, 1977). However, whereas previous studies have mainly emphasized the beneficial effect of moderate density increases, the inclusion of D3 in our study revealed a diminishing marginal increase in FIPAR. This result indicates that the response of canopy radiation interception to planting density was nonlinear within the tested density range. A plausible explanation is that excessive planting density accelerates canopy closure and intensifies shading of lower leaves, which may reduce lower-canopy photosynthetic activity and limit further improvement in effective radiation utilization (Lai et al., 2025). Reduced light availability within the canopy may also accelerate the senescence of lower leaves and constrain post-silking assimilate supply, thereby impairing grain filling and kernel weight development (Wu et al., 2024).

In the present study, F2 significantly improved RUE, with the strongest response observed under the high-density treatment D3 (**Figure 3**). Most previous studies cited above separately examined the effects of organic fertilizer on nutrient release, leaf physiological status, or soil properties, whereas our results further showed that the RUE response to organic fertilizer substitution depended on planting density. This density-dependent response suggests that partial organic fertilizer substitution may partly buffer the physiological stress imposed by dense canopies. Potential explanations include sustained nutrient release and delayed lower-leaf senescence (Fan et al., 2022; Chen et al., 2019), enhanced chlorophyll synthesis, Rubisco activity, and antioxidant protection (Gharred et al., 2022; Wang et al., 2017; Urban et al., 2021), and improvements in soil structure, microbial activity, aeration, and water-holding capacity (Guo et al., 2025). These processes could collectively support nutrient uptake and aboveground carbon assimilation.

### 4.2 Effects of planting density and organic fertilizer substitution on dry matter accumulation, translocation, and grain filling characteristics

Dry matter accumulation and remobilization constitute key physiological foundations of maize yield formation (Dong et al., 2024). Previous research has mainly documented the decline in per-plant dry matter accumulation caused by density-induced competition for radiation, water, and nutrients (Shao et al., 2024). Consistent with this general pattern, per-plant dry matter accumulation declined significantly as planting density increased in the present study (**Figure 4**). However, our study extends previous work by showing across multiple years that partial organic fertilizer substitution mitigated this decline. This finding indicates that nutrient-management strategy can modify the dry matter response of individual plants to increasing planting density, rather than merely increasing dry matter accumulation independently of density (**Figure 8**). This can be primarily explained by the ability of organic fertilizers to improve soil structure, enhance nutrient-release dynamics, and stimulate root growth, thereby increasing nutrient uptake efficiency and sustaining higher leaf photosynthetic capacity (Wu et al., 2023). Organic fertilizer has also been reported to promote the synthesis and accumulation of zeatin riboside (ZR) in roots. As a cytokinin-type hormone, ZR can delay leaf senescence by suppressing senescence-associated genes and maintaining chloroplast structural integrity (Tian et al., 2025). This pathway could have contributed to sustained photosynthetic carbon assimilation during grain filling in the present study. Further analysis revealed that pre-silking DMT under the D2F2 treatment was significantly greater than that under D1F1, with the stem making the largest contribution (**Supplementary Figure 3**, **Table 2**). The stem serves as the main reservoir of pre-silking non-structural carbohydrates (NSC) in maize, and its storage and remobilization capacity are critical for regulating grain filling (Xiao et al., 2024). In addition, about 45%–65% of the nitrogen required during grain filling is derived from the remobilization of nitrogen accumulated in vegetative organs before silking, with the stem serving as the primary organ for post-silking nitrogen remobilization (Li et al., 2024a). Furthermore, post-silking dry matter accumulation contributed more than 80% of grain dry matter accumulation (**Figure 5**), indicating that post-anthesis photosynthesis was the dominant measured source of assimilates for grain filling under our experimental conditions.

Maize yield formation fundamentally depends on the dynamic partitioning of photosynthetic assimilates, with the grain-filling stage occurring 20–40 days after silking being especially critical (Yan et al., 2024; Bonelli et al., 2016). Previous studies have mainly described how resource limitations caused by high temperature, drought, or excessive planting density shorten the active grain-filling period and alter the timing of the maximum grain-filling rate (Rattalino Edreira et al., 2014; Lizaso et al., 2017). Our results confirmed that D2 and D3 reduced both the grain-filling rate and active grain-filling duration relative to D1 (**Figure 6**, **Table 1**). More importantly, the present study further showed that 17% organic fertilizer substitution partially alleviated these density-induced constraints, particularly by improving grain-filling rate and kernel weight under dense planting. This phenomenon is mainly associated with insufficient assimilate supply from the source organs (Wang et al., 2018). Assimilate deficiency disrupts endogenous hormonal balance in grains, increasing ABA while decreasing IAA and cytokinins (CTK), thereby suppressing endosperm cell division and ultimately leading to premature cessation of grain filling (Yu et al., 2024). Our results further demonstrated that a 17% organic fertilizer substitution significantly enhanced the grain-filling rate and increased kernel weight, particularly under high planting density (**Figure 6**). The alleviation of grain-filling constraints by organic fertilizer substitution may be related to improvements in both assimilate supply and grain sink activity. Organic fertilizer has been reported to increase the activities of sucrose synthase (SuSy) and ADP-glucose pyrophosphorylase (AGPase) in grains, thereby supporting sucrose utilization and starch synthesis (Cheng et al., 2023). Organic fertilizer may also improve soil water retention by promoting macroaggregate formation and reducing soil bulk density, which could help maintain phloem transport, cellular turgor, and starch deposition during grain filling (Jiang et al., 2022; Pegler et al., 2023). Notably, random forest analysis showed that grain-filling rate had greater importance for yield than active grain-filling period (**Figure 7B**), indicating that rapid assimilate deposition during grain filling was more closely related to yield formation than the duration of the filling period. This pattern may partly reflect constraints on post-silking water and nutrient supply under high-density conditions (Gambin et al., 2023). Consequently, enhancing soil water retention and sustaining nitrogen supply after silking through integrated organic–inorganic fertilization represents a key strategy to improve grain-filling rate and facilitate yield formation.

### 4.3 Effects of planting density and organic fertilizer substitution on yield

Balancing the conflict between individual plant growth and population-level productivity is a key scientific challenge in determining optimal planting density and establishing high-yield crop populations (Shah et al., 2021). The three-year field experiment demonstrated that yield showed a unimodal response to planting density, with the highest yield observed under D2, which essentially reflects the dynamic balance between individual plant performance and population productivity. At low planting density, individual plants benefit from ample resources and space, resulting in higher kernel number and weight per plant; however, insufficient plant numbers per unit area limit overall population yield. Although aboveground dry matter accumulation per plant decreased as planting density increased from D1 to D2, population-level grain yield increased by 5.03%–25.73% (**Table 2**) because the greater numbers of plants and ears per unit area more than compensated for the reductions in individual-plant dry matter accumulation and yield components. These findings are consistent with those reported by Hernández et al. (2020). However, in 2025, when planting density was further increased to D3, intensified competition within the population led to marked decreases in kernels per ear and thousand-kernel weight, accompanied by increased barren tip length; consequently, the decline in per-plant productivity could no longer be offset by the increase in ear number, leading to reduced population yield (Gao et al., 2025). Notably, D2 (100,000 plants ha^−1^) was the highest-performing density among the treatments evaluated across all three experimental years and was higher than the optimal density of 80,000 plants ha^−1^ reported by Zhang et al. (2021) for the Loess Plateau. This suggests that the optimal planting density for maize is significantly affected by regional environmental conditions. The favorable radiation–thermal conditions and favorable soil conditions in our study area improved the population’s tolerance to high planting density. The PLS-PM results indicated that the negative association between planting density and maize yield was mainly mediated by reduced post-silking dry matter accumulation (**Figure 8**). This pathway may be associated with impaired reproductive development under high-density conditions. Previous studies have suggested that high density may prolong the anthesis–silking interval and reduce pollination success, while insufficient post-silking assimilate supply may increase ovule abortion, resulting in fewer kernels per ear and greater barren tip length and ultimately limiting grain yield (Burgess and Cardoso, 2023; Li et al., 2024b).

Furthermore, the direct and indirect effects of fertilization treatment on yield, as revealed by PLS-PM, highlight the importance of coordinating dry matter accumulation and translocation in optimizing grain filling characteristics and improving final yield. Organic fertilizer enhanced grain filling (**Figure 6**) by increasing dry matter accumulation and translocation (**Figure 5**), thereby improving yield and mitigating the negative effects associated with high planting density. A notable finding of the present study was that the 17% organic fertilizer substitution (F2) produced a greater yield advantage than the 34% substitution (F3). This nonlinear response suggests that crop performance depended not only on the amount of organic fertilizer applied, but also on the synchronization between nutrient release and crop nutrient demand. The higher substitution rate may have reduced the supply of readily available nutrients during early and middle growth stages, whereas the combined mineral and organic nutrient supply under F2 may have provided a more favorable temporal match with maize nutrient requirements (Duan et al., 2023; Zhai et al., 2022; Wang et al., 2024, 2025). Meanwhile, the benefits of organic fertilizer substitution were more evident at D2, but led to a reduction in maize yield under D3 (**Table 2**). This is likely due to the higher demand for readily available nutrients in high-density stands, while the lagged nutrient release from organic fertilizers fails to meet crop requirements during key growth stages (He et al., 2022). The promotion and application of the D2F2 strategy is expected to reduce chemical fertilizer inputs by 17% while maintaining or even increasing yield, providing significant practical value for reducing agricultural non-point source pollution and facilitating the green transformation of agriculture in semi-arid regions.

Overall, this study suggests that optimizing planting density together with partial substitution of chemical fertilizer by organic fertilizer can enhance radiation use efficiency and dry matter translocation efficiency, improve the grain-filling process, and effectively mitigate source–sink imbalance under high-density planting. Among the treatments evaluated, the D2F2 combination showed the best overall performance and may represent a promising management strategy under the conditions of this study. Nevertheless, several limitations should be acknowledged. First, the experiment included relatively few gradients of planting density and organic fertilizer substitution, and D3 was evaluated only in 2025. Consequently, the D3 response cannot be fully separated from the particular weather conditions of that year. Second, the experiment was conducted at a single location and over a limited number of years; therefore, the responses to different rainfall regimes, soil types, cultivars, and spatiotemporal variation in water and thermal resources remain uncertain (Wei et al., 2024). Third, several mechanisms proposed in the Discussion were inferred from previous literature rather than directly measured in the present experiment. Future studies should therefore combine finer density and fertilizer-substitution gradients with multi-year and multi-location field experiments. Direct measurements of vertical canopy light distribution, leaf gas exchange and chlorophyll fluorescence, soil mineral nitrogen release, root morphology, antioxidant status, endogenous hormones, and grain SuSy and AGPase activities are required to verify the proposed physiological and soil mechanisms. Integrating these measurements with crop growth models would help identify more precise and broadly applicable combinations of planting density and organic fertilizer substitution across different climatic and soil conditions.

## 5 Conclusion

This three-year field experiment examined how planting density and organic fertilizer substitution affected maize yield formation through changes in dry matter translocation and grain filling. The results showed that a planting density of 100,000 plants ha^−1^ combined with 17% organic fertilizer substitution (D2F2) performed best among the tested treatments. Compared with the conventional management practice, D2F2 increased stage-specific radiation use efficiency by 12.79%–43.62% and maize yield by 7.46%–23.82%. The PLS-PM analysis further revealed that high density primarily reduced yield by suppressing post-silking dry matter accumulation, whereas integrated organic–inorganic fertilization enhanced maize yield by stimulating pre-silking dry matter translocation and post-silking dry matter accumulation, thereby improving the grain-filling process. Overall, D2F2 may provide a promising density–fertilization management option for improving maize yield under the tested semi-arid field conditions.

## Data Availability

The original contributions presented in the study are included in the article/**Supplementary Material**. Further inquiries can be directed to the corresponding author/s.
