# Supplementary material for: Optimized planting density and organic fertilizer substitution improve maize yield by enhancing dry matter translocation and grain-filling characteristics
Source: Front Plant Sci. 2026 Aug 10;17:1913471. doi: 10.3389/fpls.2026.1913471 (PMC13500309; doi:10.3389/fpls.2026.1913471)
Supplement: Supplementary file 1 [file Supplementaryfile1.doc]

Supplementary Materials

**Optimized planting density and organic fertilizer substitution improve maize yield by enhancing dry matter translocation and grain-filling characteristics**

Liping Liu^1,2,3^，Yonghui Dai^1,2,3^，Wenlong Wang^1,2,3^，Yurong Chang^1,2,3^，Yining Ma^1,2,3^，Risheng Ding^1,2,3^, Shaozhong Kang^1,2,3^,Ling Tong^1,2,3*^

^1^ State Key Laboratory of Efficient Utilization of Agricultural Water Resources, Beijing, China

^2^ National Field Observation and Research Station (Gansu Wuwei) for Efficient Water Utilization in Oasis Agriculture, Wuwei, China

^3^ Center for Agricultural Water Research in China, China Agricultural University, Beijing, China*^[1]^*

*Corresponding author at: Center for Agricultural Water Research in China, China Agricultural University, Beijing, China.

*E-mail address: tongling2001@cau.edu.cn (L. Tong).*

**Supplementary Methods S1**. Calculation of daily incident radiation and intercepted radiation

Daily incident photosynthetically active radiation (*PAR*) was calculated from photosynthetic photon flux density (*PPFD*, μmol m⁻² s⁻¹) recorded by the on-site HOBO automatic weather station. The station recorded *PPFD* at 15-min intervals in 2023 and 2024 and at 10–15-min intervals in 2025. Daily incident *PAR* (*PAR_inc,d_*, MJ m⁻² d⁻¹) was calculated by integrating *PPFD* over each day and converting photon flux density to energy units using a coefficient of 4.57 μmol J⁻¹:

$PAR_{inc\text{，}d}=\frac{\Sigma\text{(}PPFD_{i}\times\Delta t_{i}\text{)}}{4.57\times10^{6}}$ (1)

where *PPFD_i_* is the *i*-th *PPFD* record, and Δ*t_i_* is the time interval between adjacent records (s). Because the fraction of intercepted photosynthetically active radiation (*FIPAR*) was measured only at key growth stages, daily *FIPAR* values between two adjacent sampling dates were estimated by linear interpolation.

$\begin{matrix} FIPAR_{d}=FIPAR_{i}+\frac{FIPAR_{i+1}-FIPAR_{i}}{t_{i+1}-t_{i}}\times(d-t_{i}) \\ \end{matrix}$ (2)

where *FIPAR_i_* and *FIPAR_i+1_* are the measured *FIPAR* values at sampling dates *t_i_* and *t_i_*_+1_, respectively, and *d* represents a given day between the two dates.

Daily intercepted photosynthetically active radiation (*IPAR*, MJ m⁻² d⁻¹) was then calculated as:

$IPAR_{d}=PAR_{inc\text{，}d}\times FIPAR_{d}$ (3)

Cumulative *IPAR* was obtained by summing daily *IPAR* over the corresponding growth period.

**FIGURE S1.** Schematic diagram of maize planting pattern (unit: cm).

**FIGURE S2**. Seasonal dynamics of daily and cumulative incident photosynthetically active radiation during the maize growing seasons from 2023 to 2025. Panels (a–c) show daily incident photosynthetically active radiation (PAR) in 2023, 2024, and 2025, respectively, and panel (d) shows cumulative incident PAR from 25 April to 25 September. The cumulative incident PAR values were 1377.44, 1250.92, and 1299.30 MJ m⁻² in 2023, 2024, and 2025, respectively.

**FIGURE S3.** Effects of different planting densities and fertilization treatments on the proportion of biomass partitioning to different organs per plant at different growth stages of maize in 2023 (a), 2024 (b), and 2025 (c). D1, D2, and D3 represent planting densities of 70,000, 100,000, and 130,000 plants ha⁻¹, respectively; F0, F1, F2, and F3 represent no fertilization, chemical fertilizer only, 17% and 34% organic fertilizer substitution, respectively. Growth stages include seeding, jointing, silking, filling, and maturity. Different colors represent the proportion of biomass in different organs: Ear, Grain, Sheath, Stem, Root, and Leaf. Different lowercase letters within bars indicate significant differences among treatments at the same growth stage.

**FIGURE S4.** Seasonal irrigation schedules under different planting-density and fertilization treatments during the 2023–2025 maize growing seasons. Panels (a), (b), and (c) show the irrigation schedules for the 2023, 2024, and 2025 growing seasons, respectively. Each stacked bar represents the seasonal irrigation amount for one treatment. The seven colored segments from bottom to top correspond to irrigation events 1–7, respectively, and the exact date of each irrigation event in each year is provided in the color-coded table below the panels. The numbers above the bars represent seasonal irrigation totals calculated as the sum of the seven displayed irrigation events. D1, D2, and D3 represent planting densities of 70,000, 100,000, and 130,000 plants ha⁻¹, respectively. F0, F1, F2, and F3 represent no fertilization, chemical fertilizer only, 17% organic fertilizer substitution, and 34% organic fertilizer substitution, respectively. D3 was evaluated only in 2025. Irrigation amounts for individual events were rounded to the nearest millimeter.

**TABLE S1**

Initial soil physicochemical properties and nutrient status of the experimental field before treatment application.

| Organic matter | Alkali-hydrolyzable N | Available P | Available K | Total N | Total P | Total K | pH |
| --- | --- | --- | --- | --- | --- | --- | --- |
| g kg⁻¹ | mg kg⁻¹ | mg kg⁻¹ | mg kg⁻¹ | g kg⁻¹ | g kg⁻¹ | g kg⁻¹ |  |
| 11.36 | 24.86 | 11.00 | 84.61 | 0.63 | 1.13 | 26.59 | 8.68 |

**TABLE S2**

Effects of planting density and fertilization on grain filling characteristic parameters of maize from 2023 to 2025

| Year | Treatment | Fitted equation | *R*^2^ | Grain filling parameters | | | | |
| --- | --- | --- | --- | --- | --- | --- | --- | --- |
|  |  |  |  | W_max_  /g | R_max_  /(g·d^-1^) | T_max_  /d | R_mean_  /(g·d^-1^) | AGP  /d |
| 2023 | D1F0 | y=35.29/(1+14.08e^(-0.083t)^) | 0.9789 | 17.64 | 0.73 | 31.86 | 0.40 | 72.30 |
|  | D1F1 | y=34.95/(1+17.85e^(-0.097t)^) | 0.9907 | 17.48 | 0.85 | 29.69 | 0.45 | 61.82 |
|  | D1F2 | y=37.55/(1+17.90e^(-0.096t)^) | 0.9912 | 18.78 | 0.91 | 29.91 | 0.48 | 62.21 |
|  | D1F3 | y=35.69/(1+21.50e^(-0.107t)^) | 0.9965 | 17.84 | 0.96 | 28.64 | 0.50 | 56.01 |
|  | D2F0 | y=31.68/(1+18.25e^(-0.093t)^) | 0.9900 | 15.84 | 0.73 | 31.38 | 0.39 | 64.82 |
|  | D2F1 | y=33.06/(1+21.65e^(-0.099t)^) | 0.9927 | 16.53 | 0.82 | 31.14 | 0.43 | 60.76 |
|  | D2F2 | y=33.32/(1+25.88e^(-0.110t)^) | 0.9952 | 16.66 | 0.92 | 29.60 | 0.47 | 54.58 |
|  | D2F3 | y=33.39/(1+25.36e^(-0.107t)^) | 0.9944 | 16.70 | 0.89 | 30.33 | 0.45 | 56.28 |
| 2024 | D1F0 | y=34.16/(1+25.60e^(-0.101t)^) | 0.9950 | 17.08 | 0.86 | 32.14 | 0.44 | 59.46 |
|  | D1F1 | y=37.28/(1+25.74e^(-0.102t)^) | 0.9971 | 18.64 | 0.95 | 31.82 | 0.49 | 58.78 |
|  | D1F2 | y=38.95/(1+19.76e^(-0.093t)^) | 0.9981 | 19.47 | 0.91 | 31.97 | 0.48 | 64.29 |
|  | D1F3 | y=38.78/(1+16.72e^(-0.093t)^) | 0.9965 | 19.39 | 0.90 | 30.33 | 0.49 | 64.62 |
|  | D2F0 | y=30.13/(1+36.16e^(-0.103t)^) | 0.9993 | 15.07 | 0.78 | 34.86 | 0.38 | 58.30 |
|  | D2F1 | y=36.85/(1+24.73e^(-0.090t)^) | 0.9994 | 18.42 | 0.83 | 35.52 | 0.43 | 66.43 |
|  | D2F2 | y=38.10/(1+24.15e^(-0.091t)^) | 0.9959 | 19.05 | 0.86 | 35.18 | 0.44 | 66.29 |
|  | D2F3 | y=36.70/(1+19.52e^(-0.085t)^) | 0.9947 | 18.35 | 0.78 | 34.80 | 0.41 | 70.26 |
| 2025 | D1F0 | y=35.00/(1+43.74e^(-0.092t)^) | 0.9947 | 17.50 | 0.81 | 40.91 | 0.39 | 64.97 |
|  | D1F1 | y=40.43/(1+18.46e^(-0.080t)^) | 0.9860 | 20.22 | 0.81 | 36.58 | 0.43 | 75.28 |
|  | D1F2 | y=40.52/(1+16.23e^(-0.080t)^) | 0.9931 | 20.26 | 0.81 | 34.83 | 0.44 | 74.99 |
|  | D1F3 | y=37.91/(1+23.82e^(-0.089t)^) | 0.9854 | 18.96 | 0.85 | 35.44 | 0.44 | 67.06 |
|  | D2F0 | y=30.39/(1+33.89e^(-0.089t)^) | 0.9852 | 15.20 | 0.68 | 39.44 | 0.33 | 67.16 |
|  | D2F1 | y=34.04/(1+23.70e^(-0.087t)^) | 0.9862 | 17.02 | 0.74 | 36.33 | 0.38 | 68.86 |
|  | D2F2 | y=35.08/(1+22.65e^(-0.090t)^) | 0.9892 | 17.54 | 0.79 | 34.74 | 0.41 | 66.79 |
|  | D2F3 | y=34.62/(1+29.13e^(-0.094t)^) | 0.9978 | 17.31 | 0.81 | 35.90 | 0.41 | 63.89 |
|  | D3F0 | y=29.20/(1+70.87e^(-0.109t)^) | 0.9854 | 14.60 | 0.80 | 38.91 | 0.36 | 54.80 |
|  | D3F1 | y=37.45/(1+24.98e^(-0.086t)^) | 0.9967 | 18.72 | 0.81 | 37.28 | 0.41 | 69.51 |
|  | D3F2 | y=34.78/(1+35.09e^(-0.095t)^) | 0.9909 | 17.39 | 0.83 | 37.30 | 0.41 | 62.89 |
|  | D3F3 | y=35.64/(1+28.89e^(-0.086t)^) | 0.9875 | 17.82 | 0.77 | 39.11 | 0.39 | 69.76 |

Note: W_max_: maximum grain weight; R_max_: maximum grain filling rate; T_max_: time to maximum grain filling rate; R_mean_: mean grain filling rate; P: effective grain filling period; *R*²: coefficient of determination. D1, D2, and D3 represent planting densities of 70,000, 100,000, and 130,000 plants ha⁻¹, respectively; F0, F1, F2, and F3 represent no fertilization, chemical fertilizer only, 17% and 34% organic fertilizer substitution, respectively. D, F, and D×F indicate the effects of planting density, fertilization, and their interaction, respectively. Different lowercase letters within the same year indicate significant differences among treatments (*P* < 0.05); **P* < 0.05, ***P* < 0.01, ****P* < 0.001; ns, not significant. W_max_: maximum grain weight; R_max_: maximum grain filling rate; T_max_: time to maximum grain filling rate; R_mean_: mean grain filling rate; AGP: effective grain-filling period.

**TABLE S3**

Bio-organic fertilizer application rates and chemical and total nutrient inputs under different fertilization treatments.

| Treatment | Bio-organic fertilizer application rate | Chemical N | Chemical P | Chemical K | Total N | Total P | Total K |
| --- | --- | --- | --- | --- | --- | --- | --- |
| F0 | 0 | 0 | 0 | 0 | 0 | 0 | 0 |
| F1 | 0 | 300.0 | 87.3 | 45.7 | 300.0 | 87.3 | 45.7 |
| F2 | 600 | 249.4 | 84.8 | 40.8 | 300.0 | 87.3 | 45.7 |
| F3 | 1200 | 198.7 | 82.3 | 36.0 | 300.0 | 87.3 | 45.7 |

Note: All values are expressed as kg ha⁻¹. Chemical N, P, and K represent nutrients supplied by chemical fertilizers, whereas total N, P, and K include nutrients supplied by both chemical and bio-organic fertilizers. F0, no fertilizer; F1, chemical fertilizer only; F2, 17% organic fertilizer substitution; and F3, 34% organic fertilizer substitution.
